# Assessing health-related quality of life of people with diabetes in Nigeria using the EQ-5D-5L: a cross-sectional study

**DOI:** 10.1038/s41598-023-49322-8

**Published:** 2023-12-18

**Authors:** Idongesit Linus Jackson, Abdulmuminu Isah, Abam Onen Arikpo

**Affiliations:** 1 Department of Clinical Pharmacy and Biopharmacy, Faculty of Pharmacy, University of Uyo, Uyo, 520271 Akwa Ibom State Nigeria; 2 Department of Clinical Pharmacy and Pharmacy Management, Faculty of Pharmaceutical Sciences, University of Nigeria, Nsukka, 410001 Enugu State Nigeria

**Keywords:** Metabolic disorders, Quality of life

## Abstract

Assessing the health-related quality of life (HRQoL) of people with diabetes is important to evaluate treatment effectiveness and identify interventions that would be beneficial to the patients. This descriptive cross-sectional study aimed to assess the HRQoL of people with diabetes visiting 15 community pharmacies in Akwa Ibom State, Nigeria, and to identify its determinants. The English (Nigeria) version of the EQ-5D-5L was administered to 420 eligible patients between August and September 2021. Data were analyzed with SPSS (IBM version 25.0) and presented descriptively; differences in HRQoL scores were examined using inferential statistics. Statistical significance was set at *p* < 0.05. Most participants (56.8%) were female; 193 (49.6%) were between the ages of 30 and 49. The median (interquartile range, IQR) for the EQ VAS and EQ-5D-5L index scores, respectively, were 80.0 (65.0–85.0) and 0.77 (0.62–0.90). Most participants reported problems with usual activities (52.7%), pain/discomfort (60.2%), and anxiety/depression (57.6%). The EQ VAS score and EQ-5D-5L utility index were significantly (*p* < 0.05) associated with respondents' age, marital status, work status, and personal monthly income. The HRQoL of participants was relatively high. Nevertheless, implementing strategies aimed at pain management and providing psychological support for people with diabetes in Nigeria may improve their HRQoL.

## Introduction

Diabetes is one of the fastest growing global health emergencies of the twenty-first century^1^. According to the International Diabetes Federation^1^, there were about 537 million people with diabetes globally in 2021; this figure is projected to rise to 643 million by 2030 and to 783 million by 2045, indicating a 46% global increase in prevalence. In Africa, about 24 million adults were living with the disease in 2021. This figure is projected to rise to about 55 million by the year 2045, suggesting a 134% increase, making Africa the region with the world’s fastest growing diabetes rate^1^. A meta-analysis by Uloko et al. reported a diabetes prevalence of 5.77% in Nigeria^2^. This suggests that 11.2 million Nigerians were living with the disease in 2017 based on the country’s 193.3 million population as of September 2017^3^. The south-south geopolitical zone of the country had the highest diabetes prevalence, 9.8%, according to the meta-analysis^2^.

Diabetes poses a serious global threat to the health and well-being of individuals affected, their families and nations at large^1^. People living with diabetes are at risk of developing a range of debilitating and life-threatening complications, leading to a reduced (health-related) quality of life and premature death^1^. Hays and Reeve define health-related quality of life (HRQoL) as how well an individual functions in their daily life as well as their perceived well-being in physical, mental and social health domains^4^. In contrast to the functional component of HRQoL which consists of behaviours that can be observed by others, the well-being component refers to internal, subjective perceptions like vitality, pain, anxiety, depressive symptoms, and general health perceptions^4^.

HRQoL is an important patient-reported outcome which is increasingly being used to evaluate the effect of a medical intervention on a symptom or function or a group of symptoms or functions^5^. It is a relevant input to conduct health economic evaluations and identify cost-effective interventions that lead to efficient allocation of scarce resources^6^. Assessment of HRQoL can foster patient engagement with care and enhance a patient-centered approach to treatment^5^. This is because findings from HRQoL studies can identify subgroups with relatively poor perceived HRQoL and help to guide interventions to enhance their situations and avert more serious consequences^7^. Instruments used to measure HRQoL may be specific or generic. Specific instruments focus on a particular disease, population, functional area or problem/condition, while generic instruments can be used in any population, regardless of disease or intervention^5,8^.

Generic instruments include health profiles and utility measures^8,9^. Health profiles generate scores in a variety of health domains. Utility measures incorporate preference weights or ‘utilities’—typically obtained from a representative sample of the general population—which summarize HRQoL as a single number (index score). These index scores typically range from less than 0 (where 0 is the value of a health state equivalent to dead; negative values indicate health states considered worse than dead) to 1 (the value of full health), with higher scores suggesting higher (better) health utility^8,10^. Utilities obtained from generic preference-based measures, such as the EQ-5D-5L, can be combined with survival to generate quality adjusted life years (QALYs). QALY is a metric used in health economic evaluations (cost-utility analyses) of treatment interventions^11^. Reimbursement agencies such as the National Institute for Health and Care Excellence (NICE) routinely perform cost-utility analyses for new health care interventions^12^.

Studies assessing the HRQoL of people with diabetes using the EQ-5D-5L in Nigeria are scant. Moreover, no study has been conducted in the south-south zone of the country where diabetes is most prevalent. Hence, the present study aimed to evaluate the HRQoL of people with diabetes visiting some community pharmacies in Uyo, Akwa Ibom State, Nigeria, and to explore its determinants.

## Methods

This study sought to assess the HRQoL of people with diabetes visiting 15 community pharmacies in Uyo, Akwa Ibom State, and to explore its determinants using a descriptive cross sectional study. To select the study settings, Uyo metropolis was divided into three clusters: high, mid, and low, based on population distribution. From each of these clusters, five community pharmacies were selected by convenience, for a total of 15 pharmacies. The minimum sample size required for this study was estimated to be 377 using a 95% confidence level, a 5% margin of error, and an estimated population of 20,000 (since the population of people with diabetes in Uyo metropolis is unknown). The sample size was computed with the aid of an online sample size calculator^13^. To accommodate a possible non-response of 10%, 420 respondents were targeted for the study. The estimated sample size of 420 respondents was distributed proportionately across the selected 15 community pharmacies. Adults (18 years of age or older) with type 1 or type 2 diabetes who had been using medication(s) for diabetes for at least six months were included in the study. Participants and/or their legal guardian(s) also had to have given informed consent to participate.

### Instrument for data collection

The English (Nigeria) paper self-complete five-level version of the EuroQol five-dimensional instrument (EQ- 5D-5L) was used to assess patient HRQoL. The EQ-5D-5L is a widely used generic preference-based self-reported measure of health status consisting of two parts—the descriptive system and a visual analogue scale (VAS)^10^. In the descriptive system, five dimensions of health are evaluated: mobility, self-care, usual activities, pain/discomfort, and anxiety/depression. For each of these dimensions, there are five levels of response: no problems (level 1), slight problems (level 2), moderate problems (level 3), severe problems (level 4), and extreme problems/unable to (level 5). This section of the EQ-5D-5L offers a descriptive profile that can be utilized to generate a (5-digit) health state profile. An individual in health state 12345, for instance, would have no problems with mobility, slight problems with self-care, moderate problems with performing usual activities, severe pain or discomfort, and extreme anxiety or depression^10^. The EQ-5D-5L has a total of 3 125 (i.e. 5^5^) potential unique health states. Each of these health states has a corresponding utility value or index score obtained from an existing set of country/region specific preference weights^14^. These preference weights or value sets reflect, on average, the general population’s preferences about the value of the health state i.e. how good or bad it is on a scale anchored at 1 (a state of full health) and 0 (a state equivalent to dead). Values less than 0 represent health states considered to be worse than dead^10^. The second part of the instrument—the EQ VAS—is an overall health status scale. It allows the individual to rate the condition of their health on the day of questionnaire completion from 0 (the worst imaginable health) to 100 (the best imaginable health).The EQ-5D-5L is brief, cognitively undemanding, and is available in several languages^10^.

### Data collection

People with diabetes who visited any of the study settings within the study period were approached and invited to participate in the study by the investigators or the research assistants. The status—whether they were diabetic or not—was verified by questioning individuals who presented a prescription for (an) antidiabetic medicine(s) and/or who needed any other diabetes-related services. The objectives and methods of the study were explained to potential participants, and oral informed consent sought from them and/or their legal guardian(s). Thereafter, the EQ-5D-5L was administered to those who met the inclusion criteria. Additionally, patient demographic and clinical variables (gender, age, marital status, work status, personal monthly income, enrolment in health insurance, insulin use) were obtained through self-report. Completed questionnaires were retrieved by the investigators or the research assistants after ensuring that all fields had been filled. Data collection lasted for two months (August through September 2021).

### Data analysis

Data were analyzed using SPSS version 25.0 (IBM Corporation, Armonk, NY, USA). Descriptive statistics was used to present data. The five levels of the EQ-5D-5L were dichotomized into ‘no problems’ (level 1) and ‘any problems’ (levels 2, 3, 4 and 5)^10^. A health state index score was computed from individual health state profiles using the EQ-5D-5L crosswalk value set for Zimbabwe^15^ since a value set was not available for Nigeria at the time of writing. The Zimbabwean value set was used because the health status of the Zimbabwean general population closely approximates that of the Nigerian general population. Due to the non-normal distributions of the EQ VAS and EQ-5D-5L index scores, non-parametric tests (Mann–Whitney and Kruskal Wallis) were used to examine the differences in these scores among patient sub-groups. The level of statistical significance was set at *p* < 0.05.

Approval to conduct this study was obtained from the Akwa Ibom State Health Research Ethics Committee (AKHREC/22/7//21/014). Informed consent was obtained from all subjects and/or their legal guardian(s). All methods were performed in accordance with the Declaration of Helsinki.

## Results

A total of 389 out of 420 questionnaires distributed were accurately filled and returned yielding a response rate of 92.6%. Respondents were mostly female (56.8%); almost half (49.6%) were between the ages of 30 and 49. Two hundred and twenty-nine (58.9%) were married; only 94 (24.2%) were enrolled in the National Health Insurance Scheme (NHIS). Details of respondents’ socio-demographic and clinical characteristics are presented in Table 1.

**Table 1:** Socio-demographic and clinical characteristics of respondents (N = 389).

Characteristics	Frequency	Percent
Gender		
Male	168	43.2
Female	221	56.8
Age (years)		
Less than 30	48	12.3
30–39	98	25.2
40–49	95	24.4
50–59	84	21.6
60 or older	64	16.5
Marital status		
Single	83	21.3
Married	229	58.9
Widowed/divorced	77	19.8
Work status		
Working	186	47.8
Not working	115	29.6
Retired	88	22.6
Personal monthly income (NGN)		
Less than 30,000	134	34.4
30, 000–100,000	173	44.5
Above 100,000	82	21.1
Enrollment in NHIS		
Yes	94	24.2
No	295	75.8
Insulin use		
Yes	110	28.3
No	279	71.7

*NGN* Nigerian Naira (₦); 1NGN = 412 USD (exchange rate as at September 2021; *NHIS* National Health Insurance Scheme.

### Description of patient health profile

A total of 129 (33.2%) reported having no problems with mobility, self-care, usual activities, pain/discomfort, or anxiety/depression. More than half of the respondents reported having no problems with mobility (52.4%) and self-care (63.0%). However, most of them reported having slight to extreme problems with usual activities (52.7%), pain/discomfort (60.2%) and anxiety/depression (57.6%). When stratified by age, the number of problems reported in all five dimensions was observed to increase with age (Table 2).

**Table 2:** Description of health profile of people with diabetes.

Dimension	Age (years), n (%)					Total
	< 30	30–39	40–49	50–59	≥ 60	
Mobility						
No problems	44 (91.7)	70 (71.4)	62 (65.3)	19 (22.6)	9 (14.1)	204 (52.4)
Any problems	4 (8.3)	28 (28.6)	33 (34.7)	65 (77.4)	55 (85.9)	185 (47.6)
Self-care						
No problems	45 (93.8)	76 (77.6)	71 (74.7)	35 (41.7)	18 (28.1)	245 (63.0)
Any problems	3 (6.2)	22 (22.4)	24 (25.3)	49 (58.3)	46 (71.9)	144 (37.0)
Usual activities						
No problems	42 (87.5)	62 (63.3)	63 (66.3)	15 (17.9)	2 (3.1)	184 (47.3)
Any problems	6 (12.5)	36 (36.7)	32 (33.7)	69 (82.1)	62 (96.9)	205 (52.7)
Pain/Discomfort						
No problems	39 (81.2)	57 (58.2)	47 (49.5)	10 (11.9)	2 (3.1)	155 (39.8)
Any problems	9 (18.8)	41 (41.8)	48 (50.5)	74 (88.1)	62 (96.9)	234 (60.2)
Anxiety/Depression						
No problems	38 (79.2)	57 (58.2)	51 (53.7)	13 (15.5)	6 (9.4)	165 (42.4)
Any problems	10 (20.8)	41 (41.8)	44 (46.3)	71 (84.5)	58 (90.6)	224 (57.6)

### Self-reported health status and utility valuations of respondents

The median (interquartile range, IQR) of the EQ VAS and EQ-5D-5L index scores were 80.0 (65.0–85.0) and 0.77 (0.62–0.90), respectively. Only 11 (2.8%) participants reported the best health imaginable on the EQ VAS (i.e. 100). A detailed description of the self-rated health status (EQ VAS) and utility valuations (EQ-5D-5L index scores) of respondents, stratified by age, is given in Table 3.

**Table 3:** Health indices of respondents stratified by age.

Health Index	Age (years)					Total
	< 30	30–39	40–49	50–59	≥ 60	
EQ VAS						
Mean (SD)	81.85 (15.16)	79.67 (11.27)	80.80 (12.09)	68.21 (13.44)	59.77 (15.63)	74.47 (15.52)
Minimum	25.00	35.00	40.00	40.00	30.00	25.00
Maximum	100.00	98.00	100.00	100.00	100.00	100.00
Median	85.00	80.00	80.00	70.00	60.00	80.00
25th Percentile	70.00	70.00	75.00	60.00	50.00	65.00
75th Percentile	95.00	90.00	90.00	80.00	70.00	85.00
EQ-5D-5L Index						
Mean (SD)	0.86 (0.09)	0.81 (0.13)	0.80 (0.10)	0.61 (0.23)	0.39 (0.32)	0.70 (0.25)
Minimum	0.40	0.22	0.56	-0.15	-0.15	-0.15
Maximum	0.90	0.90	0.90	0.90	0.90	0.90
Median	0.90	0.88	0.81	0.65	0.47	0.77
25th Percentile	0.87	0.72	0.71	0.53	0.20	0.62
75th Percentile	0.90	0.90	0.90	0.73	0.63	0.90

*EQ VAS* EuroQol visual analogue scale; *SD* standard deviation.

### Effect of patient socio-demographic characteristics on EQ VAS and utility valuations

As shown in Table 4, age (*p* < *0.001*), marital status (*p* < *0.001*), work status (*p* < *0.001*) and personal monthly income (*p* = *0.042*), respectively, influenced respondents’ EQ VAS scores. Similarly, age (*p* < *0.001*), marital status (*p* < *0.001*), work status (*p* < *0.001*) and personal monthly income (*p* < *0.001*) influenced EQ-5D-5L index scores.

**Table 4:** Effect of patient variables on EQ VAS and utility valuations (N = 389).

Variable	EQ VAS score		EQ-5D-5L utility	
	Median (IQR)	*P* value	Median (IQR)	*P* value
Gender				
Male	75.0 (60.0–85.0)	0.280	0.76 (0.62–0.90))	0.335
Female	80.0 (65.0–85.0)		0.77 (0.62–0.90)	
Age (yrs)				
< 30	85.0 (70.0–95.0)	**0.000**	0.90 (0.87–90.0)	**0.000**
30–39	80.0 (70.0–90.0)		0.88 (0.72–0.90)	
40–49	80.0 (75.0–90.0)		0.81 (0.71–0.90)	
50–59	70.0 (60.0–80.0)		0.65 (0.53–0.73)	
≥ 60	60.0 (50.0–70.0)		0.47 (0.20–0.63)	
Marital status				
Single	80.0 (70.0–90.0)	**0.000**	0.90 (0.74–0.90)	**0.000**
Married	80.0 (70.0–90.0)		0.80 (0.65–0.90)	
Widowed/Divorced	60.0 (50.0–75.0)		0.60 (0.37–0.65)	
Work status				
Working	80.0 (70.0–90.0)	**0.000**	0.85 (0.72–0.90)	**0.000**
Not working	80.0 (65.0–90.0)		0.81 (0.65–0.90)	
Retired	65.0 (54.3–75.0)		0.60 (0.37–0.65)	
Personal monthly income (NGN)				
< 30 000	80.0 (63.8–90.0)	**0.042**	0.86 (0.65–0.90)	**0.000**
30 000–100 000	75.0 (65.0–85.0)		0.74 (0.62–0.88)	
> 100 000	75.0 (65.0–80.0)		0.67 (0.51–0.90)	
Enrollment in NHIS				
Yes	75.0 (70.0–85.0)	0.845	0.77 (0.64–0.90)	0.415
No	80.0 (65.0–85.0)		0.76 (0.62–0.90)	
Insulin use				
Yes	75.0 (65.0–85.0)	0.314	0.77 (0.60–0.90)	0.669
No	80.0 (65.0–85.0)		0.76 (0.62–0.90)	

*NGN* Nigerian Naira (₦); 1NGN = 412 USD (exchange rate as at September 2021; *NHIS* National Health Insurance Scheme; bold values indicate statistical significance at *p* < .05.

## Discussion

To the best of our knowledge, this is the first study using the EQ-5D-5L to evaluate the HRQoL of people with diabetes mellitus visiting community pharmacies in Nigeria. The median EQ VAS score and utility valuations (EQ-5D-5L index scores) of participants in this study were relatively high. The most commonly reported problems were with usual activities, pain/discomfort and anxiety/depression. Patient age, marital status, work status and personal monthly income were associated with EQ VAS score and utility valuations among participants.

The median EQ VAS score obtained in our study is similar to that reported in Ethiopia^16^ and Saudi Arabia^17^, but higher than that reported in Iran^6^. Further, the median EQ-5D-5L utility score obtained in our study is lower than that reported in Ethiopia^16^ but higher than that reported in Iran^6^. The disparities in these HRQoL indices might be due to variations in the countries’ demographics, socioeconomic conditions and healthcare systems. Additionally, because preference weights used in the valuation of participants’ health states often reflect national or regional values, these weights, and hence EQ-5D-5L index scores (utility valuations), can vary between nations and regions^10^. The EQ VAS, which indicates the patients' perception of their health status, was rather high in the current study. This finding might be attributed to a number of factors: Patients generally have a propensity to overestimate their health, a factor that might erroneously underestimate their need for medical treatment^18^. Furthermore, participants were people with diabetes who visited community pharmacies; as a result, they were probably more ‘stable’ than some of the patients seen in endocrinology clinics. Additionally, most of the participants were less than 50 years, and a third claimed to have no problems in any of the EQ-5D-5L dimensions. According to a prior study, persons with diabetes over the age of 50 had problems more frequently than those who were 50 years or younger across all five dimensions of the EQ-5D-5L^19^.

In our study, pain/discomfort was the dimension with the most frequently reported problems, similar to prior studies^16,20–22^. The second and third most frequently reported problems were in the dimensions of anxiety/depression and usual activities, respectively. Similar findings were reported in an earlier study conducted among people with diabetes attending a tertiary hospital in south-east Nigeria^21^. In general, the number of problems our participants reported for each dimension was observed to increase with age.

Age was found to be significantly associated with EQ VAS scores and utility valuations in this study. The EQ VAS scores and utility valuations of respondents decreased with increasing age. These findings are consistent with those of earlier studies^16,21,23^. Although diabetes generally has a negative impact on HRQoL, as people age, physical and mental limitations, as well as an increase in body pain and discomfort, are more common^24–26^. A likely cause of pain and discomfort in people with diabetes, particularly in those over the age of 40 or whose disease has not been well controlled is nerve damage. Such damage may result in numbness or even pain/discomfort that interferes with usual activities^26^. Additionally, the complex lifelong management of diabetes and its chronic, progressive nature can lead to anxiety and depression over time^27^. Hence, the number of problems reported in the anxiety/depression dimension was observed to increase with increasing age. Contrary to our finding, a Canadian study found that diabetes patients' HRQoL improved with age. However, the effect of age vanished when other patient characteristics (gender, duration of diabetes, physical activity, body mass index, insulin use, ethnicity, and smoking) were taken into account^28^. Zare et al.^6^ found no statistically significant association between age and either the EQ VAS or utility valuations.

Marital status also had an impact on the EQ VAS and utility valuations of participants in this study. Participants who were widowed or divorced had worse HRQoL than those who were single or married. In contrast to our findings, however, Zare et al. did not find any statistically significant relationship between marital status and either the EQ VAS or utility valuations^6^. The relatively poor HRQoL reported by widowed/divorced participants in our study may be explained by the physical, emotional and psychological trauma typically associated with losing a partner (via death or divorce).

In this study, respondents who reported being employed had higher EQ VAS scores and utility valuations than those who reported being unemployed or retired. Similar to this, prior studies among people with diabetes found that those who were employed had much higher EQ VAS scores and utility valuations than those who were housewives, retired, or unemployed^6,16,19,22,23^. Individuals who are employed typically have better quality of life than those who are unemployed^29^. A plausible explanation for this is that people who are employed are generally more satisfied with their lives than those who are unemployed since they can afford the necessities of life as well as the resources required to effectively manage any health conditions they may have.

Interestingly, comparatively higher EQ VAS scores and utility valuations were reported by individuals with lower monthly personal incomes than those with higher monthly personal incomes. This is contrary to the findings of prior studies where participants with lower income reported worse HRQoL compared to those with higher income^17,30^. A high income has been shown to improve evaluation of life but not emotional well-being^31^. The well-being component of HRQoL comprises internal subjective perceptions such as vitality, pain, anxiety, depressive symptoms, and general health perceptions^4^. In line with our finding, Tang reported a negative correlation between income and quality of life when other variables—the love of money and job satisfaction—were controlled^32^. The hidden costs of high income might include work-related stress and work-family conflict resulting from spending more time away from family. These factors can negatively affect an individual's physical, mental, and overall quality of life^33,34^.

The following limitations should be taken into account when interpreting these findings: The findings of this study may not apply to all people with diabetes in Nigeria because they were generated from community pharmacies in a particular state. Nevertheless, the study was conducted in a region of the country where diabetes is most prevalent^2^, and compared to a prior similar study conducted in southeast Nigeria^21^, it had a relatively large sample size. Another limitation is the cross-sectional design of this study which precludes causality between patient variables and HRQoL indices. Furthermore, we did not assess the impact of variables that could affect HRQoL, such as the presence of complications or comorbidities, participants’ smoking status, or their alcohol consumption. Finally, the use of the Zimbabwean EQ-5D-5L crosswalk value sets for valuation of participants’ health states may not have reflected the true health state utilities of our study population. This is due to the fact that while Nigeria and Zimbabwe are both African nations, their health indices are not exactly the same.

## Conclusions

The HRQoL of people with diabetes surveyed at some community pharmacies in Nigeria was relatively high. The most frequently reported problems were in the dimensions of usual activities, pain/discomfort and anxiety/depression. Patient age, marital status, work status and personal monthly income were associated with EQ VAS and utility valuations among people with diabetes surveyed. Implementing strategies aimed at pain management and providing psychological support for people with diabetes in Nigeria, especially based on their demographic information may improve their HRQoL. Findings of this study might be useful for future cost–utility analyses to evaluate the effect of interventions or policies aiming at enhancing diabetes care in Nigeria.

## Data Availability

The datasets used and/or analyzed during the current study are available from the corresponding author on reasonable request.
